# PlaqueNet: deep learning enabled coronary artery plaque segmentation from coronary computed tomography angiography

**DOI:** 10.1186/s42492-024-00157-8

**Published:** 2024-03-22

**Authors:** Linyuan Wang, Xiaofeng Zhang, Congyu Tian, Shu Chen, Yongzhi Deng, Xiangyun Liao, Qiong Wang, Weixin Si

**Affiliations:** 1 Department of Cardiovascular Surgery, the Affiliated Hospital of Shanxi Medical University, Shanxi Cardiovascular Hospital (Institute), Shanxi Clinical Medical Research Center for Cardiovascular Disease, Taiyuan, 030024 Shanxi China; 2 Department of Mechanical Engineering, Nantong University, Nantong, 226019 Jiangsu China; 3 Shenzhen Institute of Advanced Technology, Chinese Academy of Sciences, Shenzhen, 518055 Guangdong China; 4 Department of Cardiovascular Surgery, Union Hospital, Tongji Medical College, Huazhong University of Science and Technology, Wuhan, 430022 Hubei China

**Keywords:** Medical image segmentation, Coronary artery plaques, Deep residual networks, Attention mechanism

## Abstract

Cardiovascular disease, primarily caused by atherosclerotic plaque formation, is a significant health concern. The early detection of these plaques is crucial for targeted therapies and reducing the risk of cardiovascular diseases. This study presents PlaqueNet, a solution for segmenting coronary artery plaques from coronary computed tomography angiography (CCTA) images. For feature extraction, the advanced residual net module was utilized, which integrates a deepwise residual optimization module into network branches, enhances feature extraction capabilities, avoiding information loss, and addresses gradient issues during training. To improve segmentation accuracy, a depthwise atrous spatial pyramid pooling based on bicubic efficient channel attention (DASPP-BICECA) module is introduced. The BICECA component amplifies the local feature sensitivity, whereas the DASPP component expands the network’s information-gathering scope, resulting in elevated segmentation accuracy. Additionally, BINet, a module for joint network loss evaluation, is proposed. It optimizes the segmentation model without affecting the segmentation results. When combined with the DASPP-BICECA module, BINet enhances overall efficiency. The CCTA segmentation algorithm proposed in this study outperformed the other three comparative algorithms, achieving an intersection over Union of 87.37%, Dice of 93.26%, accuracy of 93.12%, mean intersection over Union of 93.68%, mean Dice of 96.63%, and mean pixel accuracy value of 96.55%.

## Introduction

Coronary artery plaque, is a prevalent cardiovascular disease that cause various significant health issues. The development of these plaques leads to a narrowing of the carotid artery, which disrupts the blood supply to the brain. This can result in transient interruptions in blood flow, potentially causing unpredictable damage to the brain. Consequently, carotid plaque segmentation research is crucial for advancing carotid atherosclerosis diagnosis and treatment. Disease severity can be evaluated with greater precision by achieving precise segmentation and localization of carotid plaques, providing a foundation for more effective treatment strategies. In cardiovascular disease treatment, early identification and segmentation timely interventions, ultimately reduce disease-related mortality [1]. Consequently, this study is significant because it may enhance patient outcomes and improve the overall management of carotid atherosclerosis, thereby contributing to a healthier and more resilient population.

Medical image segmentation, which combines the power of medical imaging with advanced deep learning techniques, provides a highly effective and intuitive means of precisely delineating areas of interest, particularly in the context of lesion detection [2]. Medical image segmentation can be broadly categorized into two distinct approaches: traditional algorithm-based and deep learning-based [3]. However, when dealing with the inherent complexity of medical images, traditional segmentation methods may fall short and often require supplementary algorithms [4]. However, this can compromise the accuracy of the segmentation results. To address these challenges, a robust deep learning segmentation model based on neural networks has emerged as a superior alternative [5, 6]. These models can learn and leverage critical feature information for segmentation tasks, thereby significantly improving the accuracy and efficiency of the segmentation process. Consequently, they consistently outperformed traditional algorithms. In recent years, several prominent deep-learning segmentation methods have risen to the forefront of medical image analysis. Notably, models such as FCN, Deeplabv3, and Deeplab3plus have gained recognition for their exceptional performance and have become the go-to solutions in this evolving field [7–9]. These innovative approaches are poised to revolutionize medical image segmentation, enabling more precise diagnostics and treatment planning for a wide range of medical conditions.

In medical image segmentation, the development of a segmentation network model is a complex and multifaceted task that requires a nuanced understanding of medical images and their associated parameters [10, 11]. This underscores the need for a segmentation network that can handle the intricacies of medical imagery, while producing precise mask outputs. PlaqueNet introduced an innovative approach that employs a multi-path parallel residual optimization network for medical image feature extraction. By leveraging the multi-path parallel residual structure of ResNet, the robust deep-level information extraction capabilities can be harnessed [12, 13].

The proposed approach involves enhancing the network by incorporating a pooling mapping function along with the original parallel residual mapping function. This deepens the network’ architecture, resulting in more effective feature extraction. The pooling mapping function mitigates the feature information loss that often occurs in deeper networks, thereby preserving the overall feature information. To improve the accuracy of the segmented areas further, this study proposes implementing of a deep bicubic attention-space separable convolution module. The proposed method capitalizes on deep separable atrous convolution to capture a more comprehensive range of pixel information while avoiding information loss in the network output segment. Additionally, the bicubic attention mechanism augments the network’s capacity to identify local information, thereby enhancing the overall accuracy of the segmented regions. This study also introduces a novel technique designed to enhance the accuracy of medical image segmentation. It introduces a bilinear reflection filling upsampling network that incorporates reflection filling into the bilinear upsampling network. This, in conjunction with the depth bicubic sampling attention space separable convolution module, jointly evaluates the loss function, leading to an overall improvement in the network’s segmentation accuracy. However, the proposed algorithm has certain limitations. This research primarily focused on the segmentation of vascular plaques in two-dimensional images, neglecting the segmentation of three-dimensional images. Consequently, the process of segmentation visualization overlooks the corresponding three-dimensional information structure, and the resulting two-dimensional structure fails to capture the complete three-dimensional characteristics of the vascular plaques. Future study will focus on three-dimensional image vascular plaque segmentation.

In summary, this study made four main contributions.

1. The introduction of PlaqueNet for coronary artery plaque segmentation not only enhances the network’s feature extraction capabilities but also utilizes a joint evaluation loss function to significantly improve both efficiency and accuracy.
2. The advanced residual net (AResNet) module, which excels at extracting input feature information, ensuring the retention of global information during the depth-based feature extraction process.
3. The depthwise atrous spatial pyramid pooling based on bicubic efficient channel attention (DASPP-BICECA) module expands the perceptual field range of feature information, reinforces local information connections, and increases the sensitivity of the network to feature information.
4. The BINet module was designed to simultaneously assess network output loss and elevate the overall network’ segmentation performance.

With the increasing global prevalence of cardiovascular diseases, it is crucial to recognize that the primary catalyst underlying these conditions is the rupture of cardiovascular atherosclerotic plaques. Such ruptures can trigger a series of sudden and often devastating brain diseases with high mortality and disability rates, including strokes, cerebral infarctions, and cerebral hemorrhages [14]. The early detection of atherosclerotic plaques has the potential to significantly reduce the incidence of these brain-related diseases; and timely intervention following detection can help avert these sudden health crises [15]. Currently, deep learning-based medical and image processing techniques are key to precisely segmenting plaques. Extensive research has been conducted in the field of medical image segmentation. Xu et al. [16] proposed an automatic segmentation method for arterial vessel walls and plaques that would be beneficial for quantifying arterial morphology in magnetic resonance imaging, using the convolutional neural network VWISegNet model to extract features from MRVWI images and compute the class of each pixel to facilitate the segmentation of the vessel walls. Xu and Zhu [17] developed a semantic segmentation algorithm based on convolutional neural networks focusing on edge segmentation to segment arterial vessel walls and plaques to facilitate the quantitative assessment of plaques in patients with is chemic stroke.

A novel MSFA-U-Net segmentation method was introduced in the context of local radiotherapy for the thyroid segmentation of CT images. This method enhances the traditional U-Net model by incorporating multiple parallel channels, thereby enabling the fusion of feature information across different image resolutions. This strategic feature fusion approach prevents the generation of single-resolution information in U-Net during the downsampling process, thereby enhancing the accuracy and effectiveness of thyroid segmentation [18]. For the diagnosis and treatment of cancer, one of the key challenges lies in accurately delineating prostate sites from histopathological images obtained through cell puncturing. To address this issue, a BSP U-Net model is proposed to achieve precise prostate contour extraction. BSP U-Net builds upon the traditional U-Net network structure by incorporating prior knowledge of the prostate shape, resulting in more accurate and reliable prostate site localization [19, 20]. A pivotal step in automatic lung disease analysis is the accurate identification and segmentation of lung regions. To address this challenge, the VI-FCN algorithm was introduced to identify and segment the lung regions in frontal and lateral chest radiographs. This innovation is a critical contribution to the field of lung-disease analysis, aiding in the early diagnosis and treatment of such conditions [21]. Moreover, in the face detection domain, existing detectors often struggle to extract sufficient features, particularly from small-scale faces, which may result in missing detection data. To mitigate this issue, the R-FCN algorithm was proposed for small-scale face detection, offering a more robust solution for capturing facial features, even in challenging scenarios [22]. In diabetic retinopathy detection, which can be accurately identified through retinal fundus images, an enhanced object-detection algorithm basted on the R-FCN is introduced. This innovative approach incorporates a feature pyramid network and improved region structure, thereby bolstering the ability to recognize small-area objects with greater precision [23]. Position emission tomography imaging is one of the most effective methods for diagnosing malignant tumors. To alleviate the substantial workload on radiologists, a novel approach leveraging a multi-scale Mask R-CNN has been proposed, which significantly streamlines the diagnostic process [24]. In the domain of recognizing protein macromolecule crystallization, there has been a concerted effort to enhance the accuracy of classification algorithms. To achieve this, a groundbreaking strategy is presented: the application of the Mask R-CNN model to the detection of protein macromolecule crystallization. This innovative methodology also incorporates adaptive histogram techniques into Mask R-CNNs to mitigate issues, such as backlighting and precipitation effects, further refining the recognition process [25].

Many existing recognition algorithms overlook variations in spatial information within different perception fields. Some networks do not consider the relationships between the edge pixels in the target area, leading to misclassification and recognition errors. To mitigate this issue, the MR R-CNN addresses the problem by adjusting the step size of the region of interest alignment [26]. Deeplabv3plus is highly regarded as an exceptional segmentation algorithm in image segmentation, owing to its remarkable ability to effectively extract multi-scale information [27]. To pursue cerebrovascular and cranial nerve segmentation in medical images, an extended version of the Deeplabv3 algorithm was introduced. This extension incorporates a feature extraction module within the encoder structure and a shrinking pyramid pooling module into the decoder structure [28]. For the segmentation of glioblastoma tumor subregions, normal tissues, and the background, a novel algorithm named DeepNet was proposed. It leverages the structure of Deeplabv3plus and utilizes a predictively trained Resnet18 for weight initialization, resulting in more accurate and reliable segmentation results [29].

To circumvent the risk of early glaucoma-related visual impairment, the Deeplabv3plus architecture was harnessed for optic disc segmentation in the initial screenings with the specific aim of achieving accurate detection. This involves substituting multiple encoder modules in Deeplabv3plus with convolutional layers to enhance segmentation performance [30]. In thyroid segmentation in ultrasound images, a novel approach capitalizes on spatiotemporal recurrent deep learning networks that incorporate time series information. Specifically, it leverages an LSTM model based on Deeplabv3plus to conduct semantic segmentation, thereby facilitating the automatic identification of thyroid components [31]. For the real-time segmentation of bladder lesions in cystography, a range of neural network models were employed during the training phase. The results demonstrated that the PAN model outperformed the other models, thereby demonstrating its superior performance in this context [32]. To enhance the precision of pressure sore diagnosis and overcome the limitations of manually marking feature points in traditional machine learning, a novel superpixel-assisted classification image-labeling method rooted in a regional organization was introduced [33]. To address the need for more accurate eye detection and segmentation, an enhanced Deeplabv3plus network architecture was proposed [34]. In prostate cancer screening, where efficiency and precision are paramount, a deep learning-based approach for swift and accurate detection of abnormal cells was proposed [35]. For early pneumonia diagnosis using lung X-rays, a segmentation model based ResNet was developed to reduce the error rates associated with traditional methods [36]. In the domain of brain tumor detection, a modified ResNet architecture was presented to augment the watershed model, distinguishing it from conventional machine learning techniques [37]. Furthermore, in the pursuit of improved diagnostic tools for pneumonia detection, an automated pneumonia detection and diagnostic tool based on a pre-trained deep learning CNN architecture was introduced [38].

## Methods

The PlaqueNet architecture introduced in this study features a multi-path parallel residual network structure, complemented by a deep-pooling mapping function to enhance feature extraction. This deep-pooling mapping function was seamlessly integrated into each residual structure within the multi-path parallel residual network, thereby maintaining the integrity of the feature information during the transmission of pixel data. This approach enables the model to gain more valuable insights from the input data. In the final stages of the segmentation mask output, the innovation includes the introduction of a deep bicubic attention space separable convolution module. This module leverages deep separable dilated convolution to expand the scope of feature information capture and effectively minimize information loss. Simultaneously, the bicubic attention mechanism augments the relevance of the local information, resulting in the generation of more contiguous segmented regions. To further augment the network segmentation performance, an auxiliary prediction network loss module was introduced. This module combines the bilinear reflection-filling up sampling network with the deep bicubic attention space-separable convolution module, and collaboratively address the network’s segmentation loss function. This comprehensive approach significantly enhances the accuracy and effectiveness of the segmentation process.

### Deepwise parallel residual optimization module

**Fig. 1:**
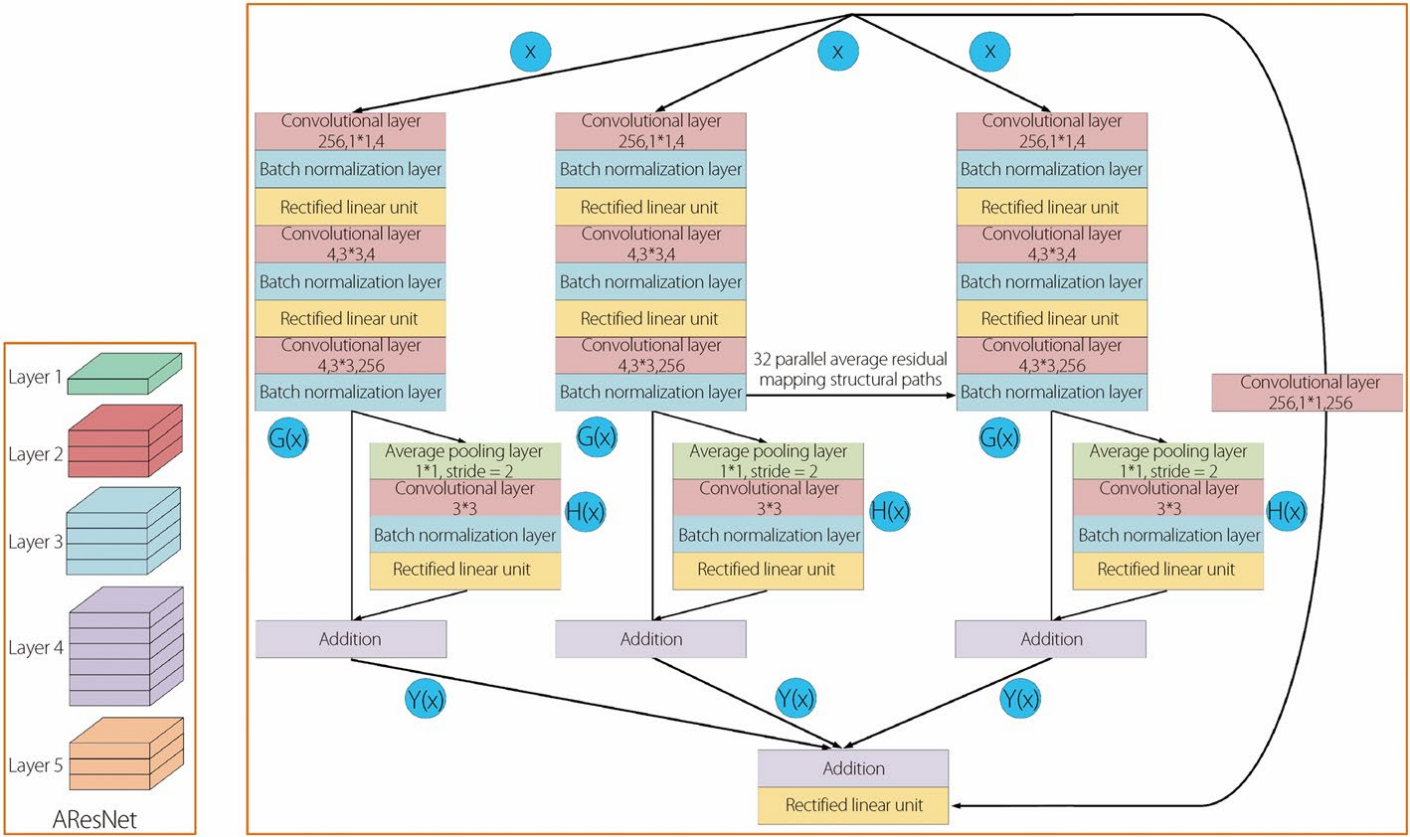
AResNet network module

 Enhancing the computational prowess of a neural network model for processing input data typically involves augmenting or modifying the depth and width of the network. However, such operations place significant demands on the network’s design and computing capacity. The Resnet network comprises a series of identical residual mapping functions structured in parallel, with all the residual blocks sharing the same topological configuration. In total, there are 32 identical residual structures. This study introduces the AResNet network, which builds on the Resnet architecture by incorporating a deep residual optimization structure within each residual mapping component (Fig. 1). This structural feature is referred to as the deep parallel residual mapping optimization function, denoted by $Y\left(x\right)$. $Y\left(x\right)$comprises two key components: the initial feature point extraction result ${G}_{i}\left(x\right)$, which is derived from the feature extraction module, and the optimization information ${H}_{i}\left(x\right)$ obtained through the deep residual optimization structure. ${G}_{i}\left(x\right)$ represents the initial outcome of the feature point extraction, whereas${H}_{i}\left(x\right)$ maps, filters, and extracts feature information from the initial residual results, thereby effectively eliminating redundant information during dimension reduction. This process ensures that edge information is accurately extracted for the target region.

The structure denoted by ${H}_{i}\left(x\right)$ comprises several key components: an average pooling layer, a convolutional layer, a batch normalization (BN) layer, and an activation function. This structural composition further extracts and optimizes feature information for each residual structure. By considering feature points from adjacent areas and computing their averages, the average pooling layer contributes to the preservation of the background in medical images. This preserved background served as a valuable reference point for comparing segmentation results and facilitating disease diagnosis. The combination of the convolutional and BN layers mitigates the training challenges associated with the depth of the residual structure. It effectively addresses issues such as gradient disappearance and explosion, which can hinder the model’s performance during the training process.

Additionally, the activation layer enhances the adaptability of the feature information, making it easier for the segmentation result to fit the data. Moreover, it helps reduce the number of model parameters, thereby optimizing the feature extraction module.

${H}_{i}\left(x\right)$ is the result of Deepwise residual optimization structure.

1 $$\begin{array}{c}H_i\left(x\right)=Relu\left(\sum\limits_{i=1}^M\left(k_{ci}x_i+k_{APi}x_i\right)+b_i\right)\end{array}$$

Where ${x}_{i}$ represents the input feature information, ${k}_{ci}$ represents the convolution kernel of the convolution layer, ${k}_{APi}$ represents the convolution kernel of the average pooling layer, ${b}_{i}$ represents the bias function, $Relu$ represents the activation function, and $M$ represents the number of convolutional layers.

${Y}_{x}$is the result of Deepwise parallel residual optimization functions.

2 $$\begin{array}{c}Y_x=\sum\limits_{i=1}^C\left(G_i\left(x\right)+H_i\left(x\right)\right)+x\end{array}$$

where${G}_{i}\left(x\right)$ represents the convolutional result in the parallel residual network structure, ${H}_{i}\left(x\right)$ represents the result of the depthwise pooling feature extraction function, and $C$ represents the cardinality in the parallel mapping residual network.

### DASPP-BICECA module

The DASPP-BICECA module plays a pivotal role in predicting the segmentation output stage. DASPP, which employs deep atrous convolution operations with varying atrous convolution rates, extends the scope of regional information perception during convolution. Deepwise separable convolution effectively segregates the regional input information from the channel convolution points, thereby reducing the number of parameters during model transmission. The integration of BICECA further enhanced the preservation of feature information throughout the sampling process, resulting in outstanding segmentation outcomes.

**Fig. 2:**
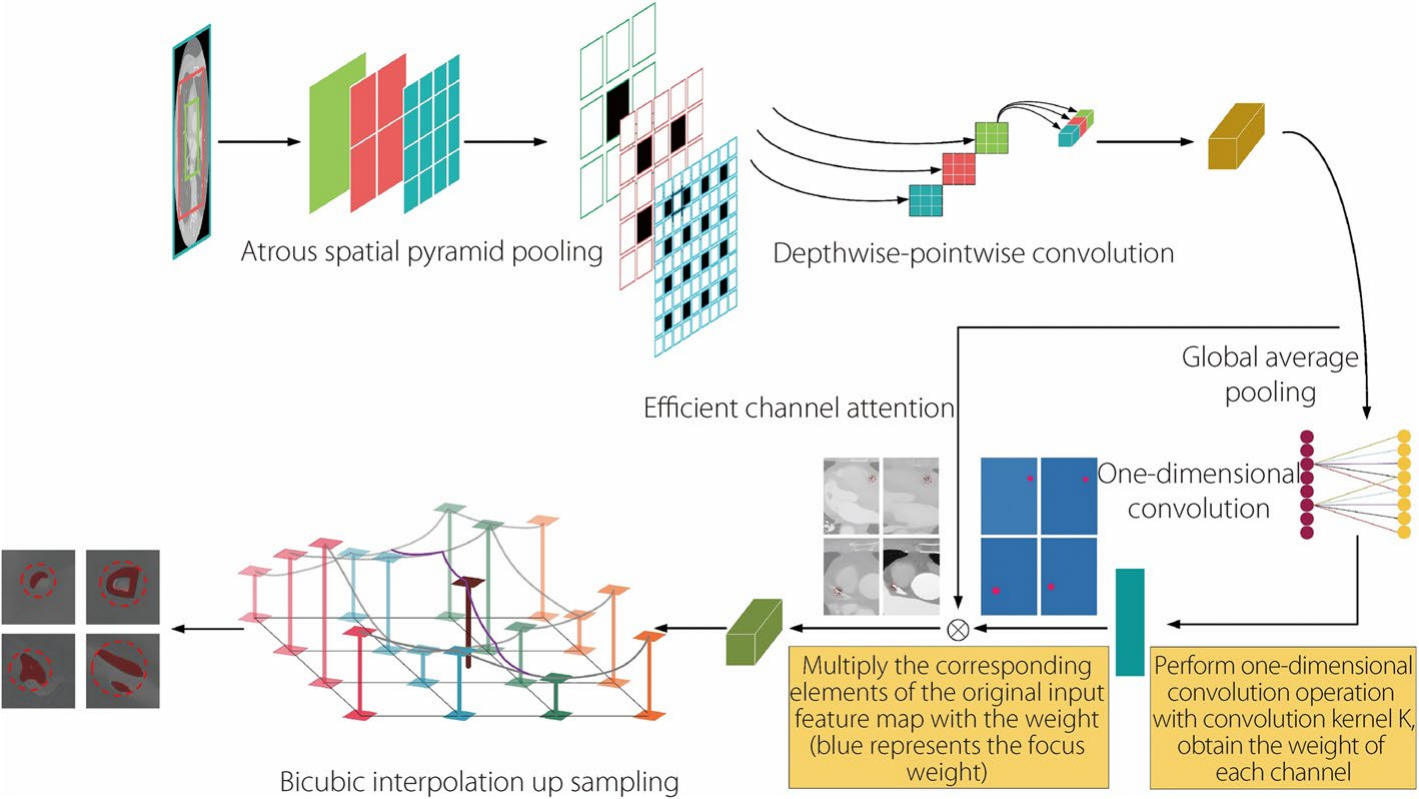
Network output prediction using the DASPP-BICECA module

 The network architecture for forecasting the output of the carotid plaque segmentation region is illustrated in Fig. 2. In this structure, the input image undergoes an initial convolution at various dilation rates through the atrous convolution layer. Subsequently, depthwise convolution and pointwise convolution employing depthwise separable convolution techniques were applied to fine-tune the model parameters. The target region is obtained through bicubic interpolation, yielding a high-resolution segmentation region.

The DASPP module reduces the number of network parameters during model training. ASPP through hole convolution effectively extends the perceptual field range by processing the input image with various atrous convolution rates. This process divided the features sampled at each unique hole convolution rate into separate branches for subsequent processing. By employing different atrous rates, the model can capture a broader context of image information while avoiding adverse effects on image resolution that may result from large step sizes during convolution. Equation 3 defines the ASPP hole pooling process, where $Q(c,d)$ represents the output outcome of the hole convolution in coordinates $(c,d)$. Given that depthwise separable convolution varies its convolution kernels based on the different channels of the input network, the convolution process is bifurcated into two segments. The depthwise convolution operation is expressed in Eq. 4, followed by the pointwise convolution operation in Eq. 5. Equation 6 encapsulates the ultimate output result of depthwise separable convolution. Here, ${DConv({\beta }_{d},Q)}_{(i,j)}$ denotes the output of depthwise convolution, ${PConv({\beta }_{p},Q)}_{(i,j)}$ stands for the output of pointwise convolution, and ${DSConv({\beta }_{d},{\beta }_{p},Q)}_{(i,j)}$ represents the output of the depthwise separable convolution.

3 $$\begin{array}{c}\textit{Q}\mathit{\left(\text{c,d}\right)}\mathit=\underset{\textit{i,j}}{\mathit\sum}\textit{q}\mathit{\left({\text{c}+\text{e}\cdot\text{i,d}+\text{e}\cdot\text{j}}\right)}\mathit\cdot\textit{k}\mathit{\left(\text{i,j}\right)}\\\end{array}$$

where $q$ is the input information, $e$ is the rate of atrous convolution, $k$ is the filter, and $\left(i,j\right)$ is the location of the atrous convolution layer where the convolution is performed.

4 $$\begin{array}{c}{DConv\left(\beta_d,Q\right)}_{\left(i,j\right)}=\sum\limits_{m,n}^{M,N}\beta_d\left(m,n\right)Q\left(i+m,j+n\right)\end{array}$$

5 $$\begin{array}{c}{PConv\left(\beta_p,Q\right)}_{\left(i,j\right)}=\sum\limits_v^V\beta_p\left(v\right)Q\left(i,j\right)\end{array}$$

6 $$\begin{array}{c}{DSConv\left(\beta_d,\beta_p,Q\right)}_{\left(i,j\right)}={PConv\left(\beta_p,Q\right)}_{\left(i,j\right)}\left(\beta_p,{DConv\left(\beta_d,Q\right)}_{\left(i,j\right)}\right)\end{array}$$

where$Q$ represents the input information; ${\beta }_{d}$ represents the convolutional layer weight of the channel convolution; ${\beta }_{p}$ represents the convolutional layer weight of the point convolution; $M$ and $N$represent the dimensions of the convolutional layer, respectively; and $V$ represents the point convolution of a channel.

To increase the model’s sensitivity to the feature points within the target region, BICECA was introduced into the prediction mask output segment. This attention mechanism safeguards critical information during the convolution process by allocating distinct weight coefficients to the input feature regions, and subsequently selecting the region information to be segmented. ECA employs dynamic convolution kernels, treating a one-dimensional convolution as a non-fully connected layer, with each convolution operation affecting only a fraction of the convolution layers.

The input data pass through a global average pooling layer with activation, converting two-dimensional convolution into a one-dimensional counterpart, as expressed in Eq. 7. The local cross-channel interaction operation of the one-dimensional convolution, detailed in Eqs. 8 and 9, combines the input information to derive the attention factor. This attention factor is then integrated with the two-dimensional input information through the activation function to yield the attention channel output, denoted by$${Q}_{(i,j)}^{\left(H*W*C\right)}$$, as shown in Eq. 10. Bicubic interpolation leverages the grayscale values surrounding the sampled pixel points for interpolation. It fits the grayscale influence of 16 neighboring pixel points in an adjacent area and determines the pixel value of the target pixel through a weighted summation of the surrounding pixel values. Equation 11 outlines the dual-cubic interpolation process, where $$G(x,y)$$ is the output of the dual-cubic linear function.

7 $$\begin{array}{c}q_{avg\left(i,j\right)}^{\left(1\ast1\ast C\right)}=\sum\limits_{i,j}{F\left(Relu\left({GAP}_{avg}\left({DSConv\left(Q\right)}^{\left(H\ast W\ast C\right)}\right)\right)\right)}_{\left(i,j\right)}\end{array}$$

8 $$\begin{array}{c}\phi_{c\left(i,j\right)}^{\left(1\ast1\ast C\right)}=\sum\limits_{i,j}\alpha{\left(LCCI\left(q_{avg\left(i,j\right)}^{\left(1\ast1\ast C\right)}\right)\right)}_{\left(i,j\right)}\end{array}$$

9 $$\begin{array}{c}\eta_{A\left(i,j\right)}^{\left(H\ast W\ast C\right)}=\sum\limits_{i,j}{\left(\phi_{c\left(i,j\right)}^{1\ast1\ast C}\otimes DSConv\left(Q\right)^{\left(H\ast W\ast C\right)}\right)}_{\left(i,j\right)}\end{array}$$

10 $$\begin{array}{c}Q_{\left(i,j\right)}^{\left(H\ast W\ast C\right)}=\sum\limits_{i,j} Relu{\left(\eta_A^{\left(H\ast W\ast C\right)}\oplus DSConv\left(Q\right)^{\left(H\ast W\ast C\right)}\right)}_{\left(i,j\right)}\end{array}$$

where $${q}_{avg\left(i,j\right)}^{\left(1*1*C\right)}$$ is the one-dimensional output after a global average pooling function, $${\phi }_{c\left(i,j\right)}^{\left(1*1*C\right)}$$ is the output after a local cross-channel interaction operation, α is the sigmoid function, $${\eta }_{A\left(i,j\right)}^{\left(H*W*C\right)}$$ expresses the attention factor, and $$Relu$$ is the activation function.

11 $$\begin{array}{c}G\left(x,y\right)=\sum\limits_i^3\sum\limits_j^3Q_{\left(i,j\right)}\delta\left(y-y_j\right)\delta\left(x-x_i\right)\end{array}$$

where ${Q}_{\left(i,j\right)}$is the input information and $\delta \left({x}_{i}\right)$ and $\delta \left({y}_{j}\right)$ are the interpolation weighting factors in the horizontal and vertical directions, respectively.

### Joint assessment of network loss module

BINet is introduced as a collaborative evaluation-loss module for PlaqueNet. It uses the feature information extracted by AResNet and employs it to predict the segmentation mask area without affecting the final model output. The difference between the predicted and actual mask areas served as the basis for calculating the loss.

**Fig. 3:**
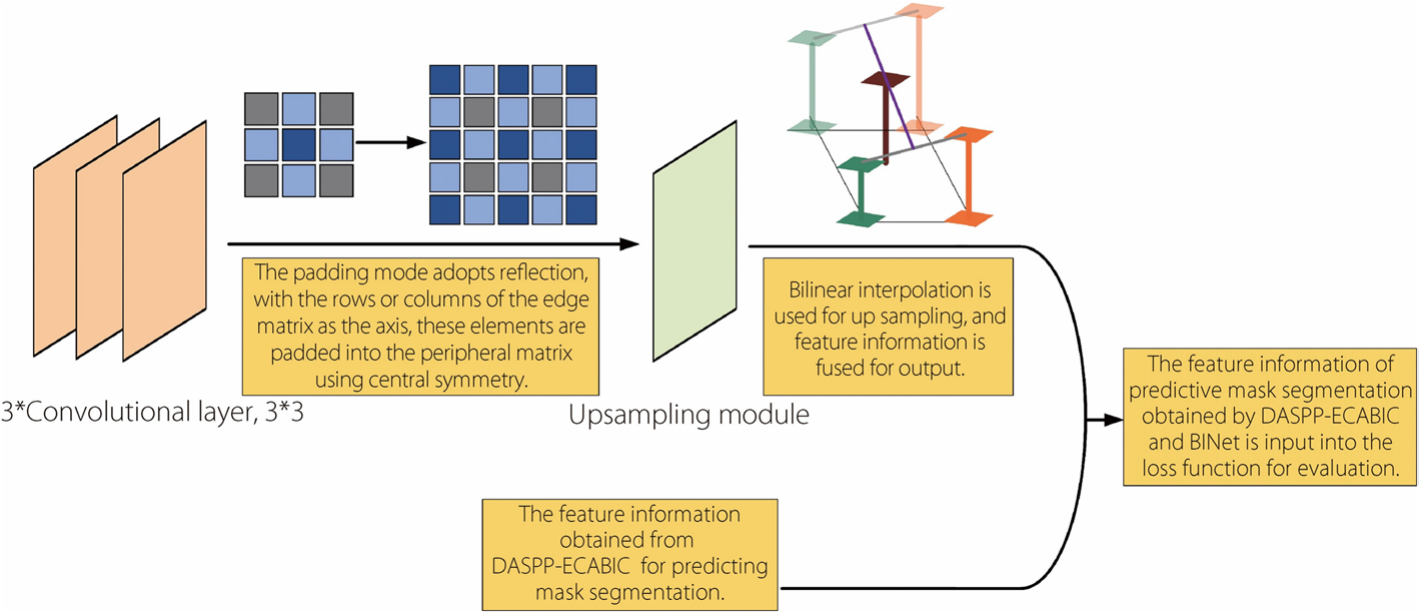
Loss of BINet network joint assessment model

 This loss value was then combined with the loss derived from the DASPP-BICECA network to form a comprehensive loss function. This refined function provides a more accurate representation of the gap between the predicted and actual values, resulting in an enhanced segmentation model, as illustrated in Fig. 3.

Equation 12 illustrates the output following the reflection-filled convolution operation, whereas Eq. 13 shows the results after the BINet upsampling of the output. Here, ${r}^{N}$denotes the output of the reflection-filled convolution layer, and ${R}^{n}$ represents the output of the upsampling module.

12 $$\begin{array}{c}r^N=\sum\limits_1^N\left(\sum\limits_i^{K-1}\sum\limits_j^{K-1}r\left(x+i,y+i\right)k_{\left(i,j\right)}\right)\end{array}$$

13 $$\begin{array}{c}R^n=F_{Avg}\left(\left(1-\alpha_x\right)\left(1-\alpha_y\right)r_{\left(m,n\right)}^{N-1}+\alpha_x\left(1-\alpha_y\right)r_{\left(m+1,n\right)}^{N-1}+\left(1-\alpha_x\right)\alpha_yr_{\left(m,n+1\right)}^{N-1}+\alpha_x\alpha_yr_{\left(m+1,n+1\right)}^{N-1}\right)\end{array}$$

where $N$ is the number of convolutional layers, $r\left(x+i,y+i\right)$ is the pixel value at $\left(x+i,y+i\right)$, and $avg$ is a pooling function.

BINet incorporates a reflective filling convolution operation combined with bilinear upsampling. This method ensures the consistency of the nearest-neighbor interpolation throughout the upsampling procedure, effectively preventing any disruptions in the prediction mask within the segmentation region.

Algorithm 1 is used as an example to offer an intuitive depiction of the process of jointly evaluating PlaqueNet’s loss function.

**Figure:**
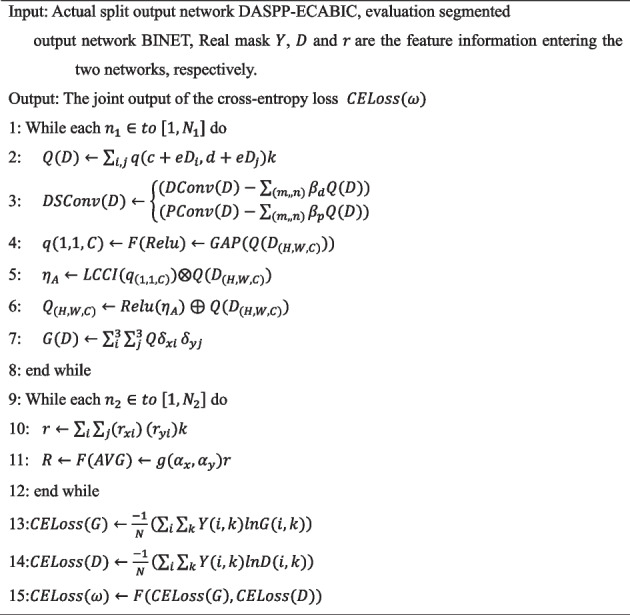
**Algorithm 1** Joint evaluation of cross-entropy loss function

## Results

The goal was to extract and convert these slices into planar images for segmentation and recognition. The research dataset included 742 images, divided into a training set of 519 images and a test set of 223 images. The PlaqueNet segmentation model we trained using these two-dimensional slice images of the vascular plaque model. In addition, three established segmentation algorithms (FCN, Deeplabv3, and Deeplabv3plus) were evaluated by considering their parameter configurations and segmentation results to assess the performance of PlaqueNet.

**Fig. 4:**
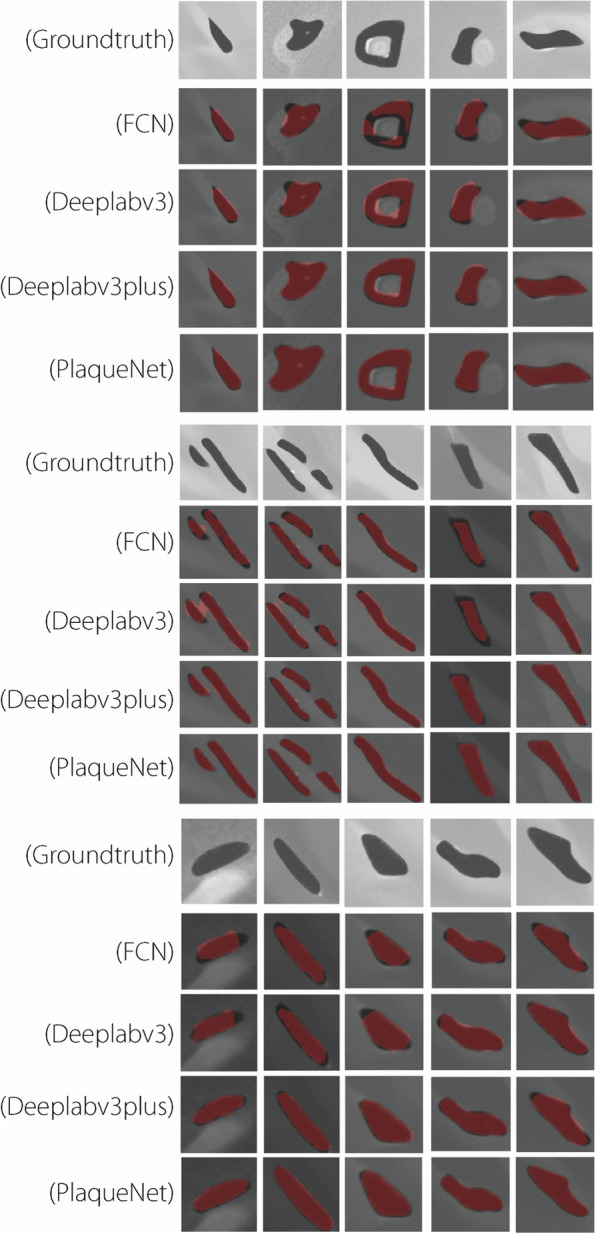
Comparison of four segmentation algorithms, each represented by a row, and the columns display the segmentation result plots. The first row corresponds to the FCN algorithm, which exhibited noticeable segmentation gaps. The second row represents the Deeplabv3 algorithm, which shown a significant over-segmentation in its results. The third row showcases the Deeplabv3plus algorithm, which demonstrates a lower degree of over-segmentation. The fourth row presents the PlaqueNet algorithm proposed in this study

 Four control experiments were conducted using the dataset presented in this study, focusing on FCN, Deeplabv3, Deeplabv3plus, and PlaqueNet. The results are shown in Fig. 4, with rows one to four representing the employed segmentation algorithms and columns one to nine displaying the outcomes produced by these algorithms. FCN’s segmentation results revealed substantial discontinuous segmentation regions. Deeplabv3 exhibits both discontinuous and over-segmented results. Deeplabv3plus’s results suffer from excessive segmentation. In contrast, PlaqueNet’s segmentation results surpassed those of the previous three segmentation algorithms. There were no discontinuous or over segmented areas, and the entire segmented region effectively covered the target area.

In this study, the segmentation results generated by the four different algorithms were evaluated using six key evaluation metrics: intersection over Union (IoU), Dice, accuracy, mean IoU (mIoU), mean Dice (mDice) and mean pixel accuracy (MPA). The Dice, which is a pixel-level similarity measure, is commonly employed to assess segmentation performance, with higher values indicating more accurate segmentation. The IoU indicates the degree of overlap between segmented and actual regions. The accuracy, a measure of model accuracy, quantifies the proportion of correct classifications in the entire dataset, offering insights into the model’s quality. The mIoU, which is the average intersection ratio, provides an overview of the IoU across the entire dataset and represent the average IoU across all categories. Similarly, the mDice was computed by averaging the Dice coefficients across all dataset categories. MPA is an improvement in pixel accuracy. It calculates the proportion of correctly classified pixels in each class, and then calculates the average of all classes. Table 1 illustrates that PlaqueNet outperformed the other three segmentation algorithms across all evaluation metrics, underscoring the enhancement in segmentation accuracy achieved by PlaqueNet.

**Table 1:** Comparison of segmentation performance of different residual networks

Algorithm	IoU (%)	Dice (%)	Accuracy (%)	mIoU (%)	mDice (%)
FCN	61.72 ± 5.24	76.33 ± 4.22	67.86 ± 7.32	80.88 ± 2.63	88.15 ± 2.11
Deeplabv3	69.04 ± 5.87	81.66 ± 6.11	77.32 ± 6.57	84.49 ± 2.94	90.83 ± 2.23
Deeplabv3plus	72.87 ± 4.49	84.31 ± 3.07	78.52 ± 4.33	86.42 ± 2.25	92.14 ± 1.54
PlaqueNet	87.37 ± 10.38	93.26 ± 8.36	93.12 ± 10.66	93.68 ± 5.20	96.63 ± 4.19

**Fig. 5:**
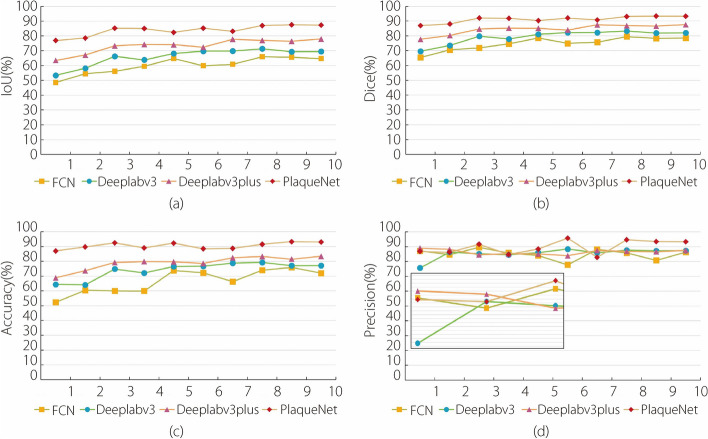
Analysis of segmentation evaluation metrics for algorithms

To further validate the segmentation performance of PlaqueNet as presented in this study, ten experiments were conducted to compare FCN, Deeplabv3, Deeplabv3plus, and PlaqueNet. The evaluation metrics included accuracy, Dice, IoU, and precision. Figure 5 clearly demonstrates that PlaqueNet’s segmentation performance surpasses that of the other three algorithms. To confirm the superior performance of the proposed AResNet structure in image segmentation compared to other residual structures, five common residual structures were selected for comparison. Their performances were evaluated using metrics such as precision, recall, F1score, and loss. As indicated in Table 2, AResNet outperformed all of the five structures.

**Table 2:** Comparison of evaluation metrics for the six segmentation algorithms

Algorithm	Precision (%)	Recall (%)	F1score (%)
ResNet	89.943 ± 5.146	76.330 ± 4.479	67.860 ± 3.075
ResNest	89.992 ± 2.875	71.399 ± 10.525	79.305 ± 8.558
ResNetVC	89.081 ± 3.944	80.586 ± 4.429	84.797 ± 3.512
ResNetVD	88.717 ± 3.944	80.185 ± 3.124	84.800 ± 3.844
ResNext	90.200 ± 3.818	70.123 ± 9.483	79.407 ± 8.896
AResNet	93.423 ± 1.936	90.623 ± 1.918	92.354 ± 1.452

**Fig. 6:**
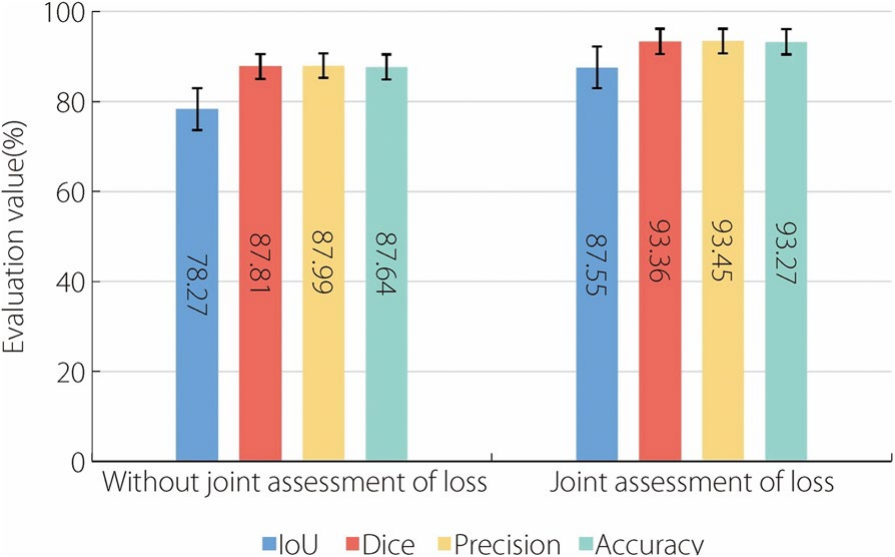
Joint assessment of the impact of network loss on segmentation performance

 To illustrate the influence of joint evaluation network loss on segmentation performance, experiments were conducted using PlaqueNet and compare its performance with and without the inclusion of joint evaluation loss. As shown in Fig. 6, the incorporation of the joint evaluation loss into the segmentation algorithm significantly improved its overall performance.

## Discussion

This study focused on the two-dimensional image segmentation of coronary artery plaques. To solve this problem, PlaqueNet, which segments coronary artery plaques from CCTA images, was introduced. In the initial stage of feature information extraction, a multi-path, parallel residual structure was introduced. This innovative structure significantly bolsters the feature extraction capacity of segmentation networks. This is accomplished by leveraging both the pooling mapping function and the original residual mapping function. Notably, this design mitigates the issue of gradient disappearance that often occurs in deep networks.

To further enhance the ability of segmentation network to capture feature information and minimize data loss, DASPP-BICECA module was presented. The BICECA component amplifies local feature sensitivity by addressing potential shortcomings of the network output segmentation, whereas the DASPP component expands the network’s information-gathering scope. Additionally, BINet for joint network loss evaluation was introduced. It optimizes the segmentation model and enhances its overall efficiency when used in conjunction with the DASPP-BICECA module.

Vascular plaques have diverse shapes and sizes across various scales. To effectively capture the feature information at different scales, a multi-scale module was employed in the algorithm used in this study. This module enables the model to accurately locate detailed information about plaques while maintaining robust segmentation performance under different scales and transformation conditions. The multi-scale module is designed to extract and incorporate features from multiple scales during segmentation. By considering information from different scales, the model effectively captures the intricate details of vascular plaques, regardless of their varying sizes and shapes. This capability enhances the ability of the model to accurately segment plaques across different scales and under different transformation conditions. The integration of the multi-scale module into the algorithm ensures that the model can effectively adapt to the variability in vascular plaque characteristics. This adaptability is essential for achieving reliable and consistent segmentation results, because plaque morphology can vary significantly across different patients, imaging modalities, and acquisition techniques. By leveraging the multi-scale module, the algorithm demonstrated improved performance in accurately locating and segmenting vascular plaques across a range of scales. These advancements have contributed to the development of more precise and clinically relevant techniques for diagnosing and treating of vascular diseases. The advantages of employing PlaqueNet for detecting coronary artery plaques are substantial. This enables the early detection of these plaques through image segmentation, thus facilitating proactive treatment and reducing the risk of cardiovascular diseases. The proposed segmentation algorithm offers precise segmentation of coronary artery plaques. This information assists healthcare professionals in evaluating disease progression and conducting personalized plaque analyses. This sets the stage for the development of more tailored clinical treatment plans.

This study has several limitations: Owing to its focus on two-dimensional image segmentation of coronary artery plaques, this approach omits critical three-dimensional structural information, which can provide a more comprehensive view of these plaques. The selected two-dimensional slices may not fully represent the entire spectrum of characteristics and intricate details of the plaques. Future studies will explore the real-time three-dimensional segmentation of medical data format images, with a specific focus on coronary artery plaques.

## Conclusions

This study introduced PlaqueNet, a novel approach to carotid plaque segmentation. PlaqueNet’s feature extraction component employs a deep parallel residual optimization mapping network that integrates a deep residual optimization structure into each residual structure in ResNet. This optimization helps maintain global information in the input feature point field, addressing issues such as gradient disappearance and explosion caused by the network depth.

The DASPP-BICECA module was used in the prediction mask output component of PlaqueNet. This module employs depth separable spatial convolution pyramid operations to expand the receptive field range of the target area during information upsampling. By initially conducting channel convolution followed by point convolution, the model training process reduces the parameter count. The BICECA module enhanced the network’s sensitivity to feature points in the target area and mitigated losses during training. Bicubic interpolation helps prevent discontinuous segmentation of adjacent feature-point areas.

Furthermore, the BINet fitting evaluation network loss module collaborates with the DASPP-BICECA module to optimize the segmentation network model. The proposed segmentation algorithm was compared with three others. The experimental results demonstrate that the proposed method achieves impressive metrics: an IoU value of 87.37%, a Dice value of 93.26%, an accuracy value of 93.12%, an mIoU value of 93.68%, an mDice value of 96.63%, and an MPA value of 96.55%. The proposed algorithm outperforms the others in terms of segmentation accuracy, avoids discontinuous or over segmented areas, and demonstrates robust segmentation performance.

## Data Availability

The raw data supporting the conclusions of this article will be made available by the authors, without undue reservation.

## Abbreviations

CCTA: Coronary computed tomography angiography
AResNet: Advanced residual net
DASPP: Depthwise atrous spatial pyramid pooling
BICECA: Bicubic efficient channel attention
BN: Batch normalization
IoU: Intersection over union
mIoU: Mean IoU
mDice: Mean Dice
MPA: Mean pixel accuracy
